# CD133-Positive Cells Might Be Responsible for Efficient Proliferation of Human Meningioma Cells

**DOI:** 10.3390/ijms13056424

**Published:** 2012-05-23

**Authors:** Hailiang Tang, Ye Gong, Ying Mao, Qing Xie, Mingzhe Zheng, Daijun Wang, Hongda Zhu, Xuanchun Wang, Hong Chen, Xiancheng Chen, Liangfu Zhou

**Affiliations:** 1 Department of Neurosurgery, Huashan Hospital, Fudan University, No.12, Middle wulumuqi road, Shanghai 200040, China; E-Mails: tangtang052105192@gmail.com (H.T.); yingmao168@hotmail.com (Y.M.); qingxie1223@sina.com (Q.X.); zhengmingzhezheng@163.com (M.Z.); wang_dai_jun@sina.cn (D.W.); zhuhongdazhu@163.com (H.Z.); xc.chen2007@yahoo.com.cn (X.C.); lfzhou@shmu.edu.cn (L.Z.); 2 Department of Endocrinology, Huashan Hospital, Fudan University, No.12, Middle wulumuqi road, Shanghai 200040, China; E-Mail: wangxuanchunwang@163.com; 3 Department of Neuropathology, Huashan Hospital, Fudan University, No.12, Middle wulumuqi road, Shanghai 200040, China; E-Mail: chenchenhonghong@126.com

**Keywords:** meningioma cells, CD133, sphere, proliferation

## Abstract

Owing to lack of appropriate model systems, investigations of meningioma biology have come to a stop. In this study, we developed a comprehensive digestion method and defined a culture system. Using this method and system, primary meningioma cells in conditioned suspension medium and a hypoxic environment could be amplified in spheres and were passaged for more than ten generations. Meningioma sphere cells were positive for meningioma cell markers and negative for markers of neural cell types. Importantly, we found the cells expressed the stem cell marker, CD133, but not nestin. All of the tumor sphere cell populations showed a slower degree of cell proliferation than that of human glioma cells and fetal neural stem cells (NSCs). Further studies showed that the proliferative rate was positively correlated with CD133 expression. The higher the CD133 expression, the faster the cell proliferation. With the increase in cell generations, the cell proliferation rate gradually slowed down, and CD133 expression also decreased. Single CD133^+^ cells rather than CD133^−^ cells could form spheres. Thus, the results above indicated that those cells expressing CD133 in spheres might be stem-like cells, which may be responsible for efficient amplification of human meningioma cells. Decreased expression of CD133 may lead to the failure of long-term passaging.

## 1. Introduction

Meningioma cell *in vitro* culture is the foundation of meningioma pathogenesis and drug treatment research [1]. So far, a stable and efficient method for meningioma cell cultures has not been established [2–5]. As we know, pre-clinical studies of meningioma depends on the *in vitro* and *in vivo* tumor model, *in vivo* models are also dependent on efficient cell models *in vitro*. However, primary and subcultured human meningioma cells are difficult, compared with other tumor cells. Therefore, establishing an efficient meningioma cell model *in vitro* becomes a necessary means for investigating the molecular basis of their growth and development, and subsequently studying meningioma pathogenesis and drug treatment. But the long-term subculture is difficult, a lot of meningioma research has been conducted mostly in primary cell-based cultures [6–8]. Although several strains have been established, except for malignant ones, most of the cell lines achieve immortalization under gene intervention, which is bound to change the characteristics of the original tumor cells [9]. Therefore, the establishment of efficient meningioma cells in primary culture and subculture systems is important for the study of the biology and development of new drug treatments.

However, it is difficult to obtain a large number of primary meningioma cells and subculture cells, due to the following factors: (1) Meningioma tissue contains a high percentage of interstitial matrix/fiber, and it is difficult to digest meningiomas using a single enzyme or mechanical methods. And long enzymatic digestion or rigid mechanical separation result in cell disruption and fewer living cells. (2) Since the majority of meningiomas are benign, cells grow slowly surrounding the tissue and cannot spread far away into the tissue. (3) A system for the cultivation of meningioma cells has not been established, and it is not clear what kind of growth factors or hormones could promote the proliferation of meningioma cells.

In a previous study, our lab established a comprehensive digestion method, which consisted of a collagenase/dispase/DNase cocktail combined with four-step digestion. Hueng *et al.* have demonstrated the existence of human meningioma cells showing stem-like features of sphere-forming ability, self-renewal, and CD133 marker [10]. And it has been demonstrated that hypoxia could enhance the generation of embryonic stem cells and induced pluripotent stem cells [11]. In addition, progesterone and androgen receptor-positive rates in meningioma tissues are about 60~90% and 65%, respectively [12,13]. A defined medium conditioned by a cell culture might contain some unknown factors, perhaps capable of stimulating its own proliferation [14–16]. To overcome those difficulties of meningioma cell culture mentioned above, in this study, we applied the comprehensive digestion method to digest meningioma tissues, then culture primary cells in the defined stem cell medium and hypoxia system. A large number of primary meningioma cells were obtained from six human meningioma tissues digested with our comprehensive digestion method. We found that these primary meningioma cells could proliferate and subculture over ten passages under sphere status in a conditioned medium and hypoxia system. Cells in sphere expressed not only epithelial membrane antigen and vimentin markers, but also CD133. Importantly, we found that the CD133 expression level was related with the cell proliferation rate. Our results suggest that stable and efficient culture systems for meningioma cells can be established under certain conditions.

## 2. Results

### 2.1. Spheres Culture of Meningioma Cells from Meningioma Tissues

Since meningiomas are difficult to digest, a suitable method was designed for digesting meningiomas. We used three enzyme cocktail recipes to digest meningiomas, combined with mechanical methods. In addition, during the digestive process, a four-step method was used to prevent cell damage caused by prolonged digestion. By using these methods, the meningioma tissue could be acutely dissociated into individual living cells.

**Figure 1:**
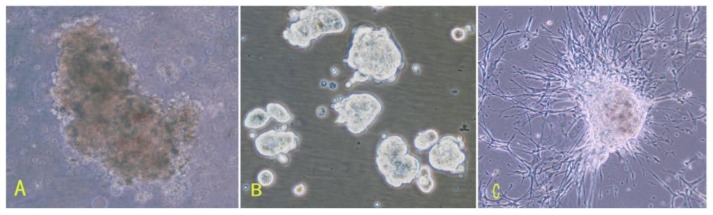

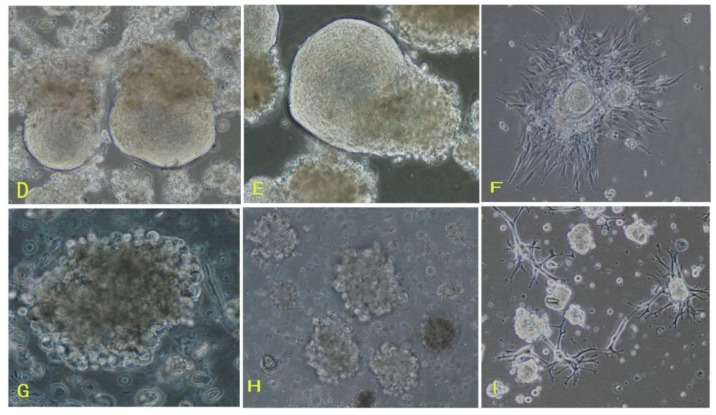
Morphologies of cells from three subtypes of meningiomas. (**A–C**) Cells from fiber meningiomas (**A**: primary spheres 200×, **B**: the third spheres 200×, **C**: adherent sphere 100×); (**D–F**) Cells from hemangiopericytoma meningiomas (D: primary spheres 200×, E: the third spheres 200×, F: adherent sphere 100×); (**G–I**) Cells from epithelial meningiomas (G: primary spheres 200×, H: the third spheres 200×, I: adherent sphere 100×).

Subsequently, cells were seeded at a concentration of 5 × 10^5^/mL. The basic culture medium was a neural stem cell medium established previously for culturing neurospheres. We added 20 nM progesterone, 100 mM androstenedione in a basic culture medium, because most kinds of meningioma express receptors for progesterone and androstenedione. Cells were cultured in a 3% O_2_, 5% CO_2_ atmosphere. The next day, cells began to gather into small spheres. Half of the old medium was replaced with fresh medium every 3 days. We named this medium a conditional medium, which consisted of half of the old culture medium and half of the fresh medium. Under the conditions described above, we established primary cultures from three meningioma subtypes (Table 1, Figure 1 A,D,G).

To determine whether primary spheres possessed the ability of self-renewal and proliferation, primary spheres were gently dissociated into single cells and maintained in the conditional medium. We found that these single cells produced secondary sphere formation after another 7–9 days, indicating that meningioma cells in spheres were capable of self-renewal and proliferation. Then, we continued to passage secondary spheres to determine whether they could form tertiary spheres, quaternary spheres and so on. We found that these sphere could be passaged for more than 10 generations (Table 1, Figure 1B,E,H). Among them, cells from two patients could be passaged for more than 15 generations. During the culture, we also found that all six samples were easy to adhere (Figure 1C,F,I).

### 2.2. Monolayer Culture of Meningioma Cells from Spheres

**Figure 2:**
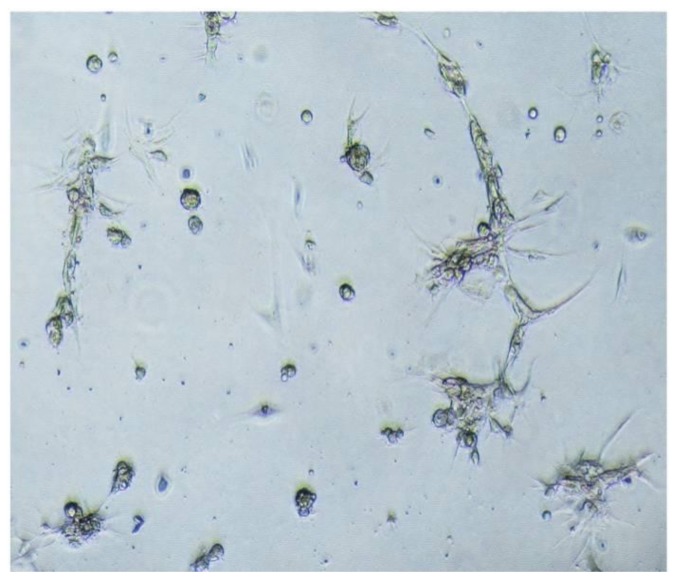
Single cells under adherent culture form vascular net-like structure (100×).

After primary meningioma cells were obtained, sphere cells were dissociated into single cells and plated in T-25 flasks according to 1:3 or 1:2 split ratios in order to observe whether meningioma cells could be cultured and passaged adherently. We found that single cells with a 1:2 split ratio became confluent within 6–7 days and could be passaged for 5–6 generations. On the contrary, single cells with a 1:3 split ratio were not easily confluent. Interestingly, we found that monolayer cells in an adherent culture could form a vascular net (Figure 2).

### 2.3. Characterization of Meningioma Cells

**Figure 3:**
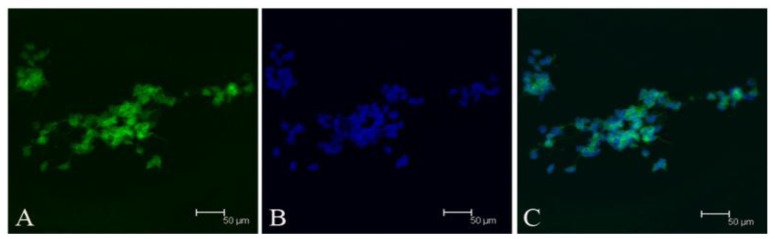
Expression of CD133 of meningioma cells. (**A**) CD133; (**B**) Hochest33342; and (**C**) merged. Scale bar = 50 μm.

**Figure 4:**
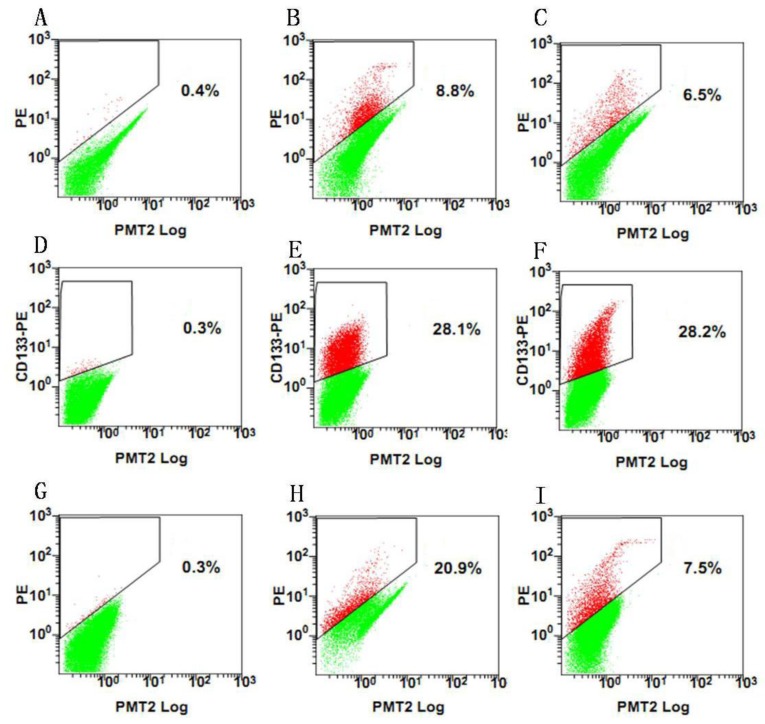
Percentage of CD133-positive cells at the third generation from different subtypes of meningioma cells. (**A**–**C**) Meningioma cells from fiber meningiomas (**A**: control, **B**: 1# patient, **C**: 2# paitent); (**D**–**F**) Meningioma cells from epithelial meningiomas (**D**: control, **E**: 5# patient, **F**: 6# paitent); (**G**–**I**) Meningioma cells from Hemangiopericytomas (**G**: control, **H**: 3# patient, **I**: 4# patient).

We first investigated whether meningioma cells expressed stem cell markers. Meningioma cells exhibited immunoreactivity for CD133 (Figure 3), and a lack of immunoreactivity for nestin (data not shown). FACS Analysis also showed there were CD133-positive cells in each subtype of meningioma cells (Figure 4). Interestingly, the percentage of CD133-positive cells in epithelial meningiomas was higher compared with that of fiber and hemangiopericytoma meningiomas (Figure 4). And the percentage of CD133-positive cells in two fiber meningiomas was the lowest among six samples, *i.e.* 8.8% and 6.5%, respectively.

**Figure 5:**
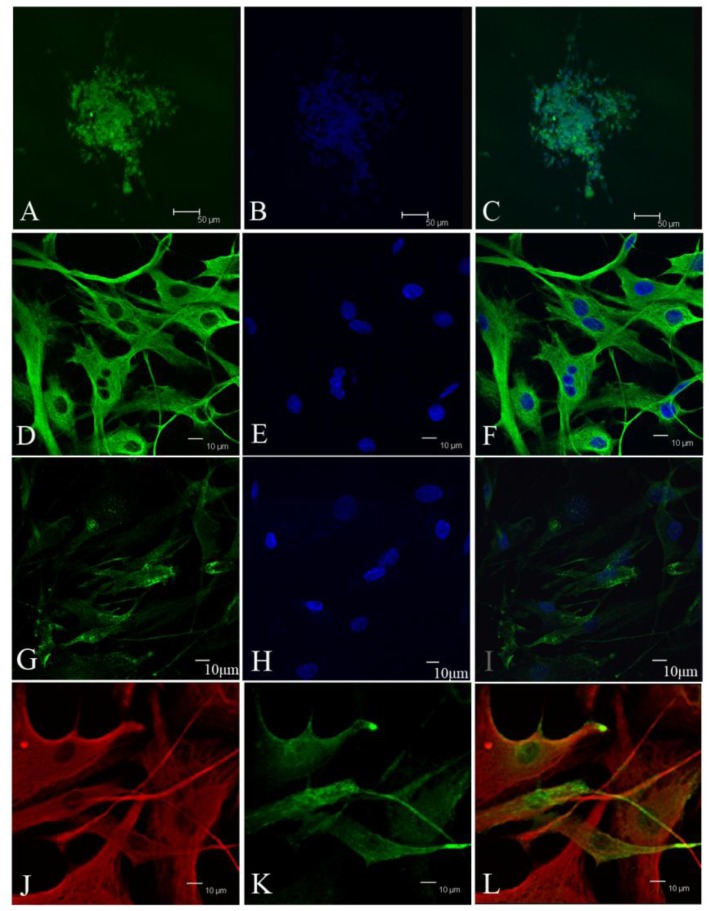
Characterization of meningioma cells. (**A**–**C**) EMA (**A**: EMA, **B**: hochest33342, **C**: merged, Scale bar = 50 μm); (**D**–**F**) vimentin (**D**: vimentin, **E**: hochest33342, **F**: merged); (**G**–**I**) Desmoplakin (**G**: Desmoplakin, **H**: hochest33342, **I**: merged). Scale bar = 10 μm. (**J**–**L**) double-labeling VEGF and vimentin (**J**: vimentin, **K**: VEGF, **L**: merged), Scale bar = 10 μm.

**Figure 6:**
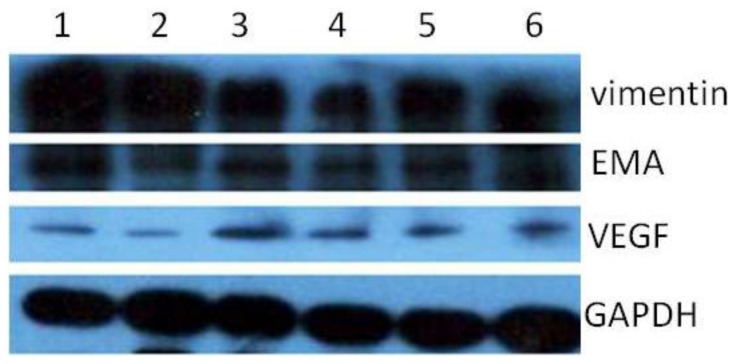
Protein expression analyzed by Western blotting. 1, 2, 3, 4, 5, 6 is the No. of the patient. Western blotting showed that each subtype of meningioma cells expressed vimentin, EMG and VEGF.

Meningioma cells of all subtypes of tumors were positive for human epithelial membrane antigen (EMA) (Figure 5A–C) and vimentin (Figure 5D–F) staining and desmoplakin (Figure 5G–I) staining, and negative for markers of neural cell types such as GFAP for astrocytes and β-tubulin III for neurons (data not showed). Since monolayer cells in an adherent culture could form vascular net, we also detected VEGF expression in double-labeling detection. A small fraction of cells in the detected cells were positive for VEGF (Figure 5J–L). Western blot analysis showed that meningioma cells of all subtypes expressed EMA, vimentin, and VEGF proteins (Figure 6).

### 2.4. Cell Proliferation Features

**Figure 7:**
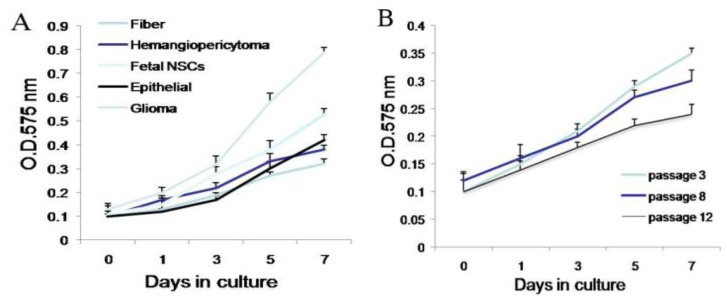

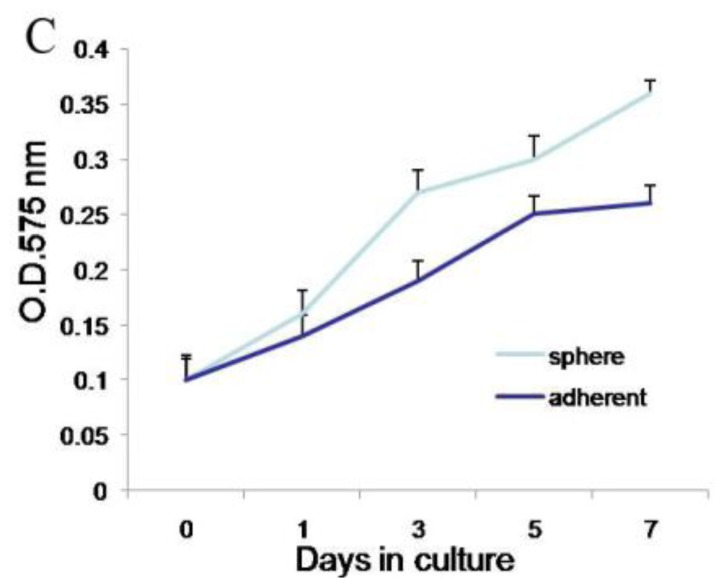
Proliferation features of meningioma cells. (**A**) Proliferation features of different subtypes of tissue-derived cells, Glioma and fetal NSCs (neural stem cells) as control; (**B**) proliferation features of the same subtypes of tissue-derived cells in different generations; (**C**) proliferation features of the same subtypes of tissue-derived cells under different culture status.

To evaluate the proliferative capacity of the tumor cells, we compared cell proliferation features of different subtypes of tissue-derived cells, the same tissue-derived cells in different culture status, and the same tissue-derived cells in different generations. Cells were plated at 1000 cells/well, and the number of viable cells was quantified on days 0, 1, 3, 5, and 7 after plating. For different subtypes of tissue-derived cells, all of the tumor sphere cell populations assayed demonstrated increased proliferative capacity, but showed a slower degree of cell proliferation than that of human glioma and fetal NSCs (Figure 7A). For the same tissue-derived cells in different culture status, cells in spheres showed a rapid proliferation, compared with cells in adherence (Figure 7B). For the same tissue-derived cells in different generations, we found that the proliferation rate slowed down with the increase of cell generations (Figure 7C).

To evaluate the proliferative capacity of CD133^+^ and CD133^−^ cells, we replated 1 CD133^+^ and CD133^−^ cell per well, respectively. After replating, only a small proportion (1–6%) of CD133^+^ tumor cells formed spheres within 48 days, whereas no *in vitro* sphere formation was observed with CD133^−^ cells (data not shown).

### 2.5. CD133 Expression Level in Meningioma Cells

**Figure 8:**
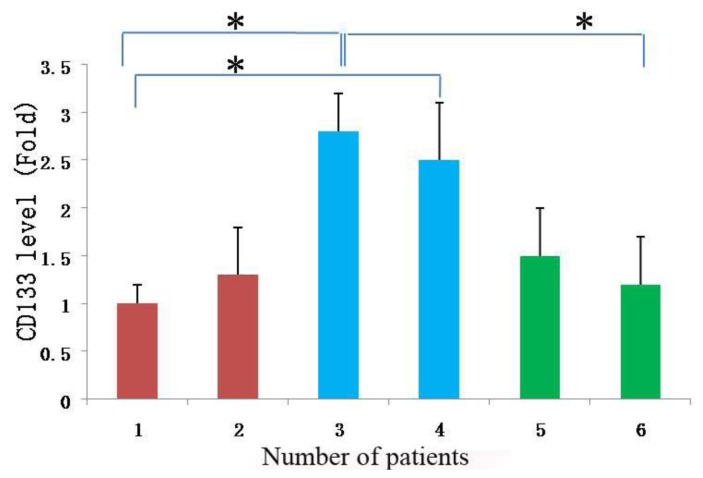
CD133 expression in meningioma cells from different subtypes of tissue. **1** and **2** (red): cells from fiber meningioma tissues; **3** and **4** (blue): cells from epithelial meningioma tissues; **5** and **6** (green): cells from hemangiopericytoma meningioma tissues. * *p* < 0.05.

**Figure 9:**
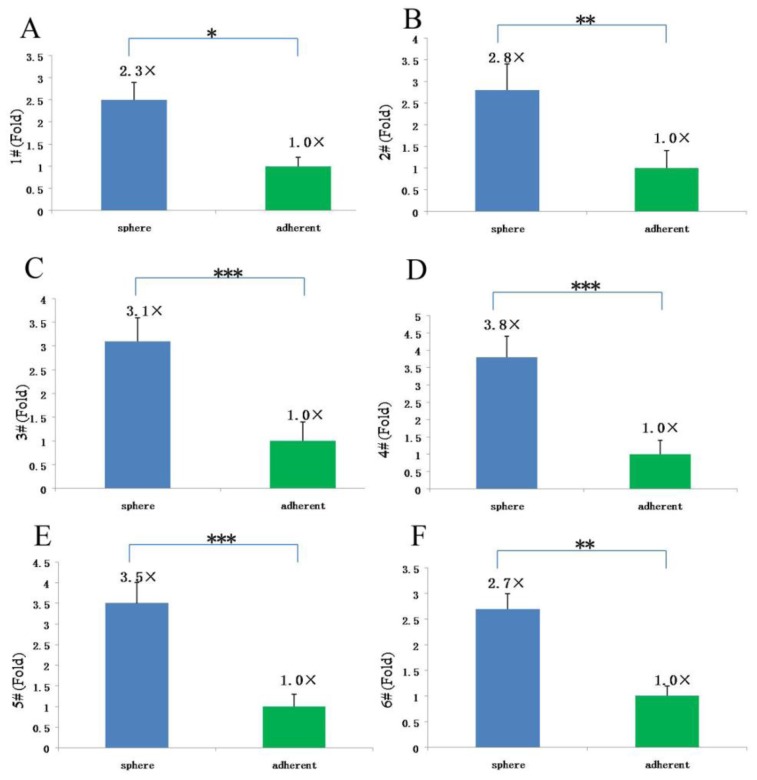
CD133 expression in the same subtypes of tissue-derived meningioma cells under different culture status. Blue: suspended sphere culture; green: adherent culture. * *p* < 0.05; ** *p* < 0.01; *** *p* < 0.001.

**Figure 10:**
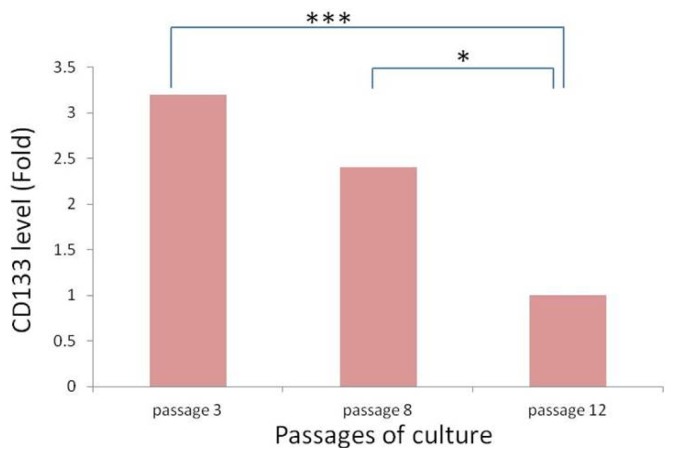
CD133 expression in different generations of the same subtypes of tissue-derived meningioma cells. * *p* < 0.05; *** *p* < 0.001.

Since cells in spheres expressed stem cell marker CD133, and cell proliferation features showed obvious differences among different subtypes of tissue-derived cells, the same tissue-derived cells in different culture status, and the same tissue-derived cells in different generations, we compared the expression of stem cell-like gene CD133 in the different meningioma cells mentioned above. The mRNA level of CD133 was assessed by quantitative RT-PCR analysis. The results showed that, for the cells from different tissues, CD133 expression in epithelial types was higher than that in fiber types (*p* < 0.05, Figure 8), but no obvious difference from hemangiopericytoma types. For the cells from the same meningioma tissue, CD133 expression in meningioma cells under sphere status was significantly higher than that under adherent status (*p* < 0.05 or *p* < 0.01, Figure 9). For the same tissue-derived cells in different generations, we found that the CD133 mRNA level decreased with the increase of cell generation, CD133 expression in the earlier generations was obviously higher than that of later generations (*p* < 0.05 or *p* < 0.01, Figure 10).

## 3. Discussion

Meningiomas have the second highest incidence in original intracranial tumors [17]. However, owing to the lack of appropriate model systems, investigations of their biology have been come to a halt. Since most meningiomas are benign, primary meningioma cells in culture are often found at senescence, resulting in the fact that studies on meningiomas had to depend on primary or early passage cells, except for those cell lines from aggressive variants of Meningiomas or immortalized cell lines. Our study showed that meningioma cells under defined conditions could be passaged over fifteen generations. Several factors below may contribute to the successful culture.

First, the comprehensive method of digestion used in this study contributed to obtaining living cells. To establish meningioma cell culture model systems, we developed a comprehensive digestion method, which consisted of three enzyme cocktail recipes and a four-step digestion method. By using the comprehensive method of digestion, we obtained a large number of living cells from each subtype of meningioma. These living cells would be easier to grow and propagate.

Second, meningioma cells under suspended spheres status might favor cell proliferation. Since meningioma cells in monolayer culture were react easily to contact inhibition and enter the quiescent and senescence states [2–5], we cultured meningioma cells under suspended spheres, thus avoiding contact inhibition, and so found that meningioma cells were not easy to enter the quiescent and senescence state. Our study showed that meningioma cells under suspended spheres could be passaged over ten generations. On the contrary, meningioma cells in adherent monolayer cultures were only passaged for four to six generations.

Third, there may be stem-like cells in meningiomas. The culture medium and environment in our study were appropriate for the growth and proliferation of stem cells. The culture medium in our study was conditional NSCs medium containing progesterone and androstenedione, and the culture system was a hypoxia system. The NSCs medium in our study is suitable for stem cells culture. Our results suggested that there would be stem-like cells in meningiomas, which is consistent with recent the identification of tumor stem-like cells as tumor-initiating cells in meningiomas [10]. In addition, a lot of studies have proved that hypoxia could enhance the generation of embryonic stem cells and induce pluripotent stem cells [11]. Therefore, we used NSCs medium as the basic medium for culturing meningioma cells in a hypoxic environment. In addition, so far, it is not clear what kind of growth factors can promote the proliferation of meningioma cells. However, defined medium conditioned by meningioma cells *per se* might contain some factors, perhaps identical to a normal endogenous growth factor *in vivo*, capable of stimulating the proliferation of meningioma cells [14]. And the progesterone and androgen receptor-positive rate in meningioma tissues were relatively high [12,13], so progesterone and androstenedione in the culture medium might contribute to the growth and proliferation of meningioma cells.

Since our results indicate that there may be stem-like cells in suspended sphere, we further investigated the expression level of CD133 of cells from different subtype tissues, different culture status, and different generations. For the same subtype of cells in different generations, the expression of CD133 of the earlier generation was obviously higher than that of the later generation. During the passage culture, we found a decreasing tendency of cell proliferation with increasing cell generation, suggesting that this decreasing tendency may be related with a decline of CD133 expression. Interestingly, cells from two epithelial subtypes could be passaged up to 15 generations, however, cells from fiber subtypes were only passaged up to 10 generations. Accordingly, CD133 expression in epithelial subtypes was higher than that in fiber subtypes, further suggesting there was a correlation between CD133 expression and cell proliferation rate. For the same subtype of cells under different status, we found that cells under suspension could be passaged for more generations, compared with those in adherent culture. Accordingly, the expression level of CD133 in cells under suspended spheres was significantly higher than that under adherence. Finally, single CD133^+^ cells rather than CD133^−^ cells could form spheres. All of this indicated that CD133-positive cells might be responsible for efficient proliferation of human meningioma cells

During the passage culture, we found that some meningioma cells could form a vascular net. Immunocytochemistry detection demonstrated that there was VEGF expression in some of the cells. Relationships between stem cells and vascular cells suggested that these VEGF-positive cells might derive from CD133-positive cell transdifferentation [18,19].

In this study, we also transplanted meningioma cells into animal models to recapitulate the original tumor. However, we did not observe the tumor (data not shown). We speculated that stem cells in meningioma tissues might only have the characteristics of tissue stem cell-like cells. Therefore, meningioma cells in our study only passaged for limited generations, similar to adult neural stem cells.

In conclusion, our study demonstrated that meningioma cells could be amplicated and passaged under sphere status and conditional system without gene intervention, but the proliferative rate declined with cell generation and decreasing CD133 expression. In our latest study, we obtained a number of clinical specimens (including three fiber meningiomas, two epithelial meningiomas, two hemangiopericytom meningiomas). The growth characteristics and trends of meningioma cells from these specimens were similar to the corresponding subtypes of this study. Therefore, CD133-positve cells might be responsible for the proliferation, and decrease of CD133 level might be one of main causes for the decline of proliferative rate.

## 4. Experimental Section

### 4.1. Ethics

The informed consents from all participants involved in this study were obtained verbally. All procedures were approved by Human Research Subjects of Huashan Hospital, Fudan University, Shanghai, China.

### 4.2. Isolation of Meningioma Cells from Meningioma Tissues

Tumor samples from six human meningioma patients were removed by craniotomy, stored in a medium in a sterile test tube and transported to the lab at 4 °C. Tumor tissues were washed ten times with pre-cooled PBS, and cut into pieces. Tissue pieces were put into a 15 mL tube, washed in PBS and centrifuged for 5 min at 500 × g. Aspirate supernatant was digested in PBS-buffered collagenase/dispase cocktail, which consisted of 1 mg/mL collagenase, 2 mg/mL dispase, 70 U/mL DNase, 0.1% BSA. Digestion was performed for 4 × 20 min in a 37 °C rotator oven (four-step method). After the first 20 min, samples were gently triturated several times with 5 mL stripette (Corning Incerperated), and settled for 5 min. The digested top tissue mixture was transferred into a new tube to centrifuge and collect cells. Some fresh collagenase/dispase cocktail was added into the remaining tissue pieces and digest was continued for the second, third and forth 20 min. During each 20 min, the manipulation described for the first 20 min was repeated.

### 4.3. Primary Tumor Spheres Culture

Cells were seeded at a concentration of 5 × 10^5^/mL into substrate-free tissue culture flasks and culture in a 3% O_2_, 5% CO_2_ atmosphere. The growth medium consisted of DMEM/F12 (phenol red-free) containing 50 units/mL penicillin–streptomycin, B27 (1:50), human recombinant FGF-2 and EGF (both at 20 ng/mL), 20 nM progesterone, 100 mM androstenedione and heparin (5 μg/mL).

### 4.4. Passaging Sphere Culture

Passaging was carried out using TrypleTM Express (Invitrogen, Carlsbad, CA, USA) and 70 U/mL DNase. When the majority of spheres were loose, 5 mL PBS were added and gently triturated, centrifuged and the cells collect at 300 g for 5 min. Cells were seeded in conditioned medium, which consisted of 1/2 of old medium and 1/2 of fresh medium. During passaging culture, 1/2 of the medium was changed every 3 days to keep it conditioned at all times.

### 4.5. Adherent Culture of Meningioma Cells

Spheres were triturated into single cell suspension, plated in T-25 flasks with a volume of 5 mL of conditional medium with 10% FBS. The growth medium consisted of DMEM/F12 containing 50 units/mL penicillin–streptomycin, B27 (1:50, Gibco, Grand Island, NY, USA), human recombinant FGF-2 and EGF (both at 20 ng/mL), 20 nM progesterone, 100 mM androstenedione, heparin (5 μg/mL), and 10% fetal bovine serum (FBS). The cells were passaged according to split ratios: 1:3 or 1:2.

### 4.6. Cell Proliferation Assays

Cells were plated in 96-well plates in 100 μL volumes of growth medium, at a density of 1000 cells/well. Cell proliferation assays were performed on days 0, 3, 5, and 7 post plating using the Cell Proliferation Kit I (MTT). Cells were incubated with MTT solution for approximately 4 h. The dye was quantified at 575 nm with an ELISA plate reader.

### 4.7. FACS Analysis

Tumor spheres were digested into single cells. The cell suspension was centrifuged at 300 g for 5 min. The supernatant was aspirated completely and the cells were pre-incubated with 20 μL of Affinity Purified Human FcγR-binding inhibitor per 100 μL Flow Cytometry Staining buffer (FCS buffer) for 20 min on ice prior to staining. Ten microliters of PE-labeled Anti-CD133 antibody (Miltenyi Biotec, Inc.) was added, mixed well and incubated for 30 min in the dark in a 4 °C refrigerator. Cells were washed by adding 2 mL of FCS buffer and centrifuged at 300 g, 4 °C for 5 min. This was repeated for a total of two washes, discarding supernatant between washes. The stained cells were resuspended in FCS buffer. Data was acquired on a FACSCalibur machine (BD Biosciences). After sorting, 1 CD133^+^ and CD133^−^ were replated into each well to evaluate the proliferative capacity of CD133^+^ and CD133^−^ cells.

### 4.8. Immunocytochemistry

Tumor sphere were partially digested into cells and loose spheres. Then these cells and loose spheres were replated on gelatin-coated glasses and cultured for 24 h. Fixed cells with 4% paraformaldehyde for 5 min at room temperature. After washing three times with PBS, the cells were treated with 0.25% Triton X-100, 10% normal donkey serum (Jackson), and 1% bovine serum albumin (BSA, Sigma) for 10 min at room temperature. Primary antibodies included CD133 (ab19898, abcam), vimentin (v6630, sigma), EMA (Dako, Carpinteria, CA, USA), Desmoplakin (ab109445, abcam), Nestin (Chemicon), bIII-tubulin (CB412, Chemicon), glial fibrillary acidic protein (Z0334, Dako). Secondary antibodies used were Alexa488-conjugated donkey anti-rabbit IgG (Invitrogen), Alexa555-conjugated donkey anti-mouse IgG (Invitrogen). The counterstain of nucleuses was 1 mg/mL Hoechst 33342 (Invitrogen).

### 4.9. qRT-PCR Analysis

The methods have been described previously [15]. Total RNA was prepared from cells using the Absolutely RNA Nanoprep Kit (Stratagene,). The forward and reverse primers of CD133 were 5′-TCT CTA TGT GGT ACA GCC G-3′, 5′-TGA TCC GGG TTC TTA CCT-3′. Complementary DNA synthesis and quantitative analysis were performed with AffiniScriptTM Muti Temperature cDNA Synthesis Kit and Brilliant II Fast SYBR Green QPCR Master Mix (Stratagene) following the manufacturer’s instructions. Amplification was performed using the ABI 7500 Real Time System.

### 4.10. Western Blot Analyses

Tumor spheres of each sample were collected for extracting protein. Western blot was performed with conventional methods [16]. Protein samples were separated with 15% SDS-polyacrylamide gel and transferred onto PVDF membranes (Millipore). The primary antibodies used in this study include CD133, EMA, vimentin, VEGF. Blots were washed and incubated for 1 h with IRDyeTM800 conjugated anti-goat and anti-mouse second antibodies.

### 4.11. Statistical Analysis

Data were analyzed using GraphPad-Prism software (version 5.0; Graphpad software Inc.: San Diego, CA, USA) and Windows XP Excel 2007 (version 2007; Microsoft: Redmond, WA, USA, 2007). Statistical analysis was performed using Student’s *t* test for mRNA level analysis. Probabilities (*p* value) < 0.05 were regarded statistically significant.

## Figures and Tables

**Table 1:** Summary of patient population.

Patient #	Sex	Age (yrs)	Pathological Subtype	Passages
1	M	69	Fiber	10
2	F	38	Fiber	10
3	M	52	Recurrence epithelial	15
4	F	44	Epithelial	15
5	M	45	Hemangiopericytoma	12
6	F	40	Hemangiopericytoma	10
